# The [FeFe] hydrogenase of *Nyctotherus ovalis *has a chimeric origin

**DOI:** 10.1186/1471-2148-7-230

**Published:** 2007-11-16

**Authors:** Brigitte Boxma, Guenola Ricard, Angela HAM van Hoek, Edouard Severing, Seung-Yeo Moon-van der Staay, Georg WM van der Staay, Theo A van Alen, Rob M de Graaf, Geert Cremers, Michiel Kwantes, Neil R McEwan, C Jamie Newbold, Jean-Pierre Jouany, Tadeusz Michalowski, Peter Pristas, Martijn A Huynen, Johannes HP Hackstein

**Affiliations:** 1 Department of Evolutionary Microbiology, Faculty of Science, Radboud University Nijmegen, Toernooiveld 1, NL-6525 ED Nijmegen, The Netherlands; 2 Nijmegen Centre for Molecular Life Sciences (NCMLS) and Center for Molecular and Biomolecular Informatics, CMBI 260 Radboud University Nijmegen Medical Centre, PO Box 9101, NL- 6500 HB Nijmegen, The Netherlands; 3 The Institute of Rural Science, University of Wales Aberystwyth, Llanbadarn Fawr, Aberystwyth, Ceredigion, SY23 3AL, Wales, UK; 4 INRA, UR1213 Herbivores, Theix, F-63122 St Genès Champanelle, France; 5 Kielanowski Institute of Animal Physiology and Nutrition, Polish Acedemy of Sciences, Instytucka 3, P-05-110 Jablonna, near Warsaw, Poland; 6 Institute of Animal Physiology, Slovak Academy of Sciences, Soltesovej 4 SK-040 01 Kosice, Slovakia; 7 RIKILT – Institute of Food Safety, Wageningen UR, The Netherlands; 8 Intervet International, Boxmeer, The Netherlands

## Abstract

**Background:**

The hydrogenosomes of the anaerobic ciliate *Nyctotherus ovalis *show how mitochondria can evolve into hydrogenosomes because they possess a mitochondrial genome and parts of an electron-transport chain on the one hand, and a hydrogenase on the other hand. The hydrogenase permits direct reoxidation of NADH because it consists of a [FeFe] hydrogenase module that is fused to two modules, which are homologous to the 24 kDa and the 51 kDa subunits of a mitochondrial complex I.

**Results:**

The [FeFe] hydrogenase belongs to a clade of hydrogenases that are different from well-known eukaryotic hydrogenases. The 24 kDa and the 51 kDa modules are most closely related to homologous modules that function in bacterial [NiFe] hydrogenases. Paralogous, mitochondrial 24 kDa and 51 kDa modules function in the mitochondrial complex I in *N. ovalis*. The different hydrogenase modules have been fused to form a polyprotein that is targeted into the hydrogenosome.

**Conclusion:**

The hydrogenase and their associated modules have most likely been acquired by independent lateral gene transfer from different sources. This scenario for a concerted lateral gene transfer is in agreement with the evolution of the hydrogenosome from a genuine ciliate mitochondrion by evolutionary tinkering.

## Background

Hydrogenosomes are membrane-bounded organelles of anaerobic unicellular eukaryotes that produce hydrogen and ATP. These elusive organelles were discovered in trichomonad flagellates, and eventually identified in quite a number of only distantly related unicellular anaerobes such as flagellates, amoeboflagellates, chytridiomycete fungi and ciliates [1-9]. Hydrogenosomes are phylogenetically related to both mitochondria and the various rudimentary, "mitochondrial-remnant" organelles collectively called "mitosomes" [7,9]. The latter organelles are found in organisms previously considered devoid of mitochondria, that were once named "archaezoa" by Cavalier-Smith [10], although one of them, *Trichomonas vaginalis*, actually was already known to contain a hydrogenosome.

The hydrogenosomes of the anaerobic ciliate *Nyctotherus ovalis *possess a mitochondrial genome and parts of an electron-transport chain on the one hand, and a hydrogenase on the other hand [11,12]. Because of this combination of features they cannot be classified as being either a hydrogenosome or a mitochondrion. It is likely that this organelle evolved from a ciliate mitochondrion by the expression of a hydrogenase that enables the ciliate to use protons as electron acceptors in order to maintain its metabolic homeostasis under anaerobic conditions. A crucial aspect of this hypothesis is the evolutionary origin of the *N. ovalis *hydrogenase itself. This is still a matter of debate since phylogenetic analyses suffer from a lack of statistical support due to an insufficient sampling of hydrogenases [13-16].

Here we present evidence that the [FeFe] hydrogenase of *N. ovalis *does not belong to the clade of "ancient eukaryotic" hydrogenases that also include the non-hydrogen producing NARF's, (nuclear prelamine A recognition factors, [17]). The analysis of the H-cluster of 19 novel hydrogenases from rumen ciliates that were recovered in a metagenomic approach, reveals the existence of another clade of [FeFe] hydrogenases from both bacterial and eukaryotic organisms, including the one of *N. ovalis*, but excluding hydrogenases from other ciliates and eukaryotes.

The [FeFe] hydrogenase of *N. ovalis *is unique because, by a fusion with two NADH dehydrogenase subunits, it is predicted to be capable of reoxidizing NADH directly. The two accessory domains responsible for this are homologous to the 24 kDa and 51 kDa subunits of the mitochondrial NADH dehydrogenase (complex I) and to the bacterial "small hydrogenases" *hoxF *and *hoxU *[11,16,18]. Supporting the origin of the hydrogenase by Horizontal Gene Transfer we show here that the accessory domains are not closely related to the *N. ovalis *complex I subunits, but rather appear to have been acquired by lateral gene transfer from bacterial ancestors that possess a [NiFe] hydrogenase.

## Results and Discussion

### The 24 kDa/NuoE/hoxF – and 51 kDa/NuoF/hoxU – like regions of the [FeFe] hydrogenase polyprotein

**Figure 1:**
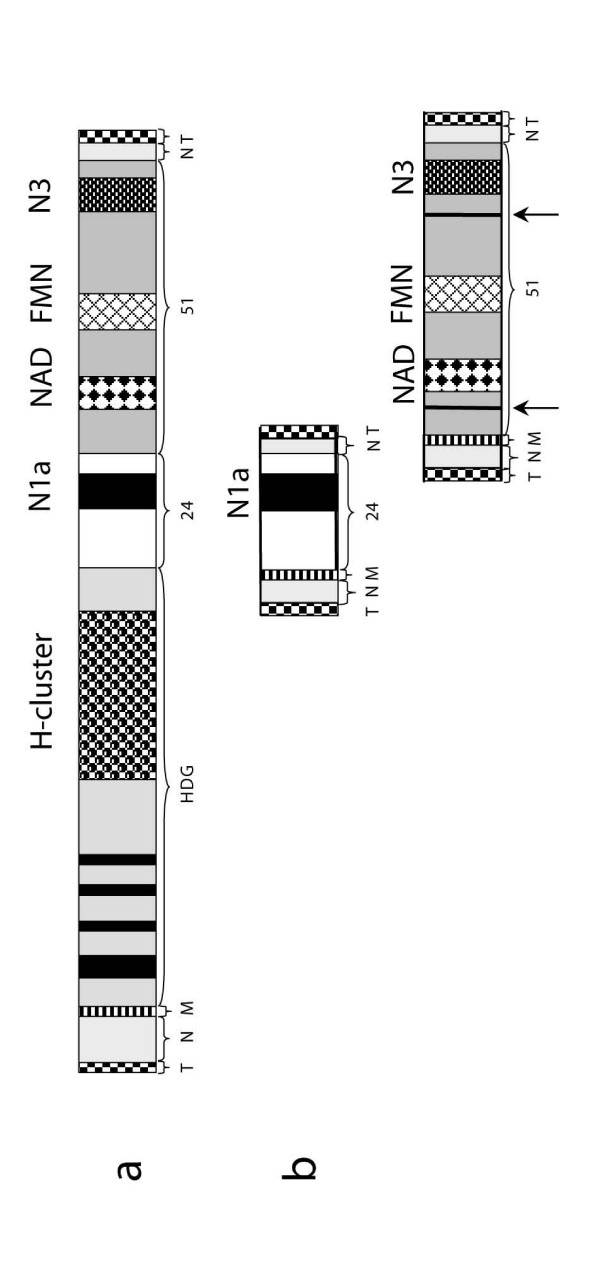
Schematic representation of the minichromosomes encoding the hydrogenase (a) and the "mitochondrial" 24 and 51 kDa genes (b). The macronuclear minichromosomes are capped by telomeres (T) and contain non-coding DNA sequences (N) at the N- and C-terminal parts of the chromosome. A mitochondrial targeting signal (M) is found at the N terminal part of the coding sequence. 1. a. The hydrogenase is chimeric, i.e. it consists of a long-type [FeFe] hydrogenase with 4 FeS clusters (black bars in HDG), a 24 kDa (hoxF) module ("24") with an N1a type FeS cluster, and a 51 kDa (hoxU) ("51") module with a N3-type [4Fe-4S] cluster plus a FMN and a NAD binding site. 1. b. The subunits of the "mitochondrial" complex I are localized on individual minichromosomes. They each possess a mitochondrial targeting signal (M) and upstream and downstream non-coding DNA (N). The "mitochondrial" 51 kDa module possesses two small introns (arrows) that are absent from the correspondent hydrogenase module.

The hydrogenase of *N. ovalis *is a polyprotein, consisting of a long-type [FeFe] hydrogenase and two (C-terminal) modules with similarity to the 24 kDa (NuoE) and 51 kDa (NuoF) subunits of complex I of mitochondrial and eubacterial respiratory chains (Fig. 1a) [13,14,18,19]. Complex I, the NADH-quinone oxidoreductase, consist of 14 subunits in eubacteria and of up to 46 subunits in (human) mitochondria [20,21]. It catalyzes the electron transfer from NADH to the quinone pool through a series of redox centers. The 24 and 51 kDa subunits are two important modules of the hydrophilic (soluble) NADH dehydrogenase part of mitochondrial complex I. The 51 kDa subunit contains a [4Fe-4S]-cluster (also known as "N3") and binding sites for NADH and FMN. The 24 kDa subunit contains a [2Fe-2S]-cluster ("N1a") [22,23].

In *N. ovalis *two different types of 24/51 kDa genes are found: (i) a hydrogenase variant, in which both subunits are fused with each other and with a [FeFe] hydrogenase, and (ii) a "mitochondrial" variant, in which the 24 kDa and 51 kDa genes are located on separate minichromosomes (Fig. 1b). As usual for *N. ovalis *and some other ciliates, the genes are located on single gene containing macronuclear minichromosomes that are capped with telomeres, making it unlikely that the genes are a contamination. Consistent with their putative function in the "mitochondrial" (hydrogenosomal) complex I (see below), these genes possess N-terminal leader sequences that likely function as a mitochondrial targeting signal. In contrast, the hydrogenase consists of a fusion of the hydrogenase, the 24 kDa and the 51 kDa subunits. Obviously, this "operon" encodes a polyprotein, since it is located on a single minichromosome, and, notably, it possesses only one (N-terminal) "mitochondrial" targeting signal (Fig 1). In contrast, both "mitochondrial" 24 kDa and 51 kDa possess their individual mitochondrial targeting signal. In addition, the "mitochondrial" 51 kDa variant contains two small introns (not shown) that are absent in the fused variant.

A multiple sequence alignment of the "mitochondrial" complex I subunits and 24 kDa/hoxF and 51 kDa/hoxU -like sequences of the hydrogenases of several *N. ovalis *species reveals that the hydrogenase modules are more similar to the *nuoE *and *nuoF *genes of a bacterial complex I than to a mitochondrial complex I (Supplementary Material). The 24 kDa-like module of the *N. ovalis *hydrogenase possesses only three of the four conserved cysteine residues that bind the [2Fe-2S] cluster N1a found in both mitochondrial 24 kDa subunits and bacterial NuoE's. The fourth cysteine residue of the hydrogenosomal [2Fe-2S] cluster has been replaced consistently by a tryptophane in all *N. ovalis *24 kDa subunits sequenced (Additional File 1). Stereochemical considerations and mutagenisation studies in bacterial *nuoE *genes have suggested that this C/W replacement most likely does not interfere with the ferredoxin-like function of the hydrogenosomal 24 kDa module [24]. The 51 kDa-like region of both the hydrogenase domain and the putative mitochondrial complex I subunits contain a NADH binding domain with four conserved glycine residues. In addition, also a FMN binding site with its conserved glycine and proline residues, and the four conserved cysteine residues of the [4Fe-4S] cluster N3 are found in both the 51 kDa subunits of mitochondrial complex I and its bacterial NuoF homologues (Fig. 1b; supplementary material).

**Figure 2:**
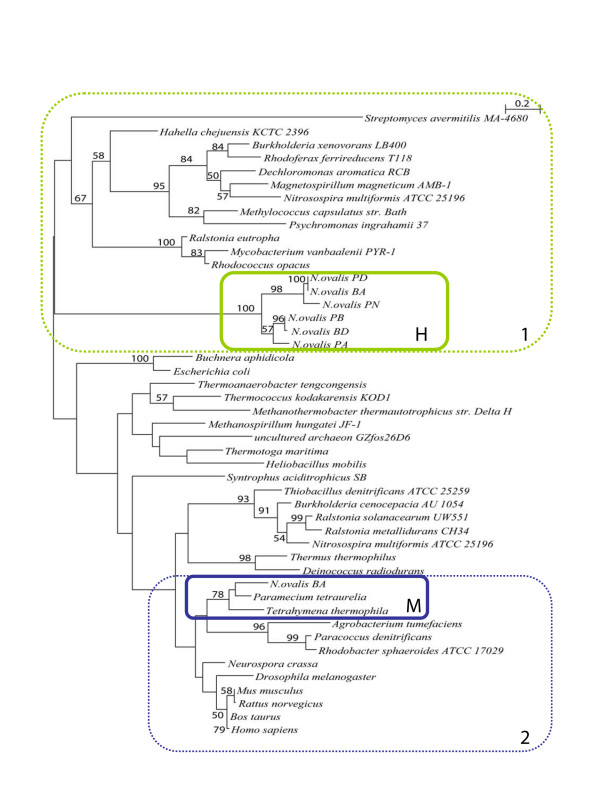
Phylogenetic tree of the 24 kDa-like module of the hydrogenase of *N. ovalis*, mitochondrial complex I 24 kDa subunits, bacterial NuoE, and bacterial hydrogenase subunits. See methods for the Accession Numbers and how the tree was calculated. H: *N. ovalis *hydrogenase, M: ciliate mitochondrial. Bootstraps are only indicated in the tree if they are ≥ 50. Box 1 marks 24 kDa modules that are fused with their corresponding 51 kDa modules (with the exception of *Nitrosospira multiformis*). All bacteria in this box (with the exception of *Nitrosospira multiformis*) have a [NiFe] hydrogenase. The mitochondrial/alpha-proteobacterial 24 kDa modules are not fused with their 51 kDa counterparts (Box 2).

Phylogenetic analysis of the 24 kDa-like region of the *N. ovalis *hydrogenase is hampered by a lack of data, especially from ciliates and other protozoa. Nevertheless, it shows clearly that this module of the *N. ovalis *hydrogenase has a bacterial rather than a mitochondrial ancestry (Fig. 2). The 24 kDa-like module from the hydrogenase clusters with its homologues from *beta*- and *gamma*-proteobacteria and the *hoxF *subunits of soluble NAD-reducing [NiFe]-hydrogenases of *beta*-proteobacteria, and not with its mitochondrial paralogues. All 24 kDa-like genes belonging to this clade are fused with their corresponding 51 kDa modules supporting the assumption of a close phylogenetic relation (with the exception of *Nitrosospira multiformis*). The *mitochondrial *24 kDa (NuoE) subunit of *N. ovalis*, on the other hand, clusters closely with its homologues from aerobic, mitochondriate ciliates. This clade belongs to a mitochondrial/*alpha*-proteobacterial sample of sequences that are only distantly related to 24 kDa sequences from *beta*- and *gamma*- proteobacteria and archaebacteria. None these sequences is fused with a *51 kDa *gene.

**Figure 3:**
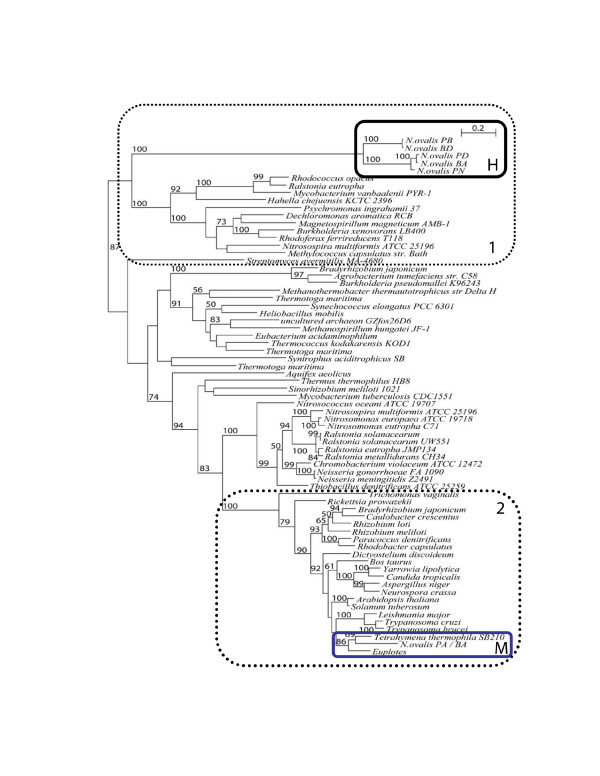
Phylogenetic tree of the 51 kDa-like module of the hydrogenase of *N. ovalis*, mitochondrial complex I 51 kDa subunits, bacterial NuoF, and bacterial hydrogenase subunits. See methods for how the tree was calculated. H: *N. ovalis *hydrogenase, M: ciliate mitochondrial. Only bootstraps ≥ 50 are indicated in the tree. Box 1 marks the fused modules (with the exception of *Nitrosospira multiformis*), Box 2 the non-fused modules of mitochondrial and alpha-proteobacterial origin. All bacteria in Box 1 (with the exception of *Nitrosospira multiformis*) have a [NiFe] hydrogenase.

Similar to the phylogeny of the 24 kDa modules, phylogenetic analysis of the 51 kDa module of the hydrogenase of *N. ovalis *shows that it is closely related to the *hoxF *subunits of soluble NAD-reducing [NiFe]-hydrogenases of beta-proteobacteria such as *Rhodococcus opacus *and *Ralstonia eutropha*, and the fused variants from *beta*- and *gamma*-proteobacteria (Fig. 3). The hydrogenase module of *N. ovalis *is more distantly related to the *nuoF *and *51 kDa *genes of the various representatives of *alpha*-proteobacterial or mitochondrial complex I genes. In contrast, the mitochondrial 51 kDa subunit of *N. ovalis *clusters with high support with its homologues from aerobic (mitochondriate) ciliates and many other eukaryotes and *alpha*-proteobacteria. It is also clearly distinct from the corresponding NADP/formate reducing hydrogenases of certain archaea and alpha proteobacteria (Fig. 3). These observations unequivocally exclude a mitochondrial or *alpha*-proteobacterial origin of the 24 and 51 kDa modules of the hydrogenase of *N. ovalis*.

**Figure 4:**
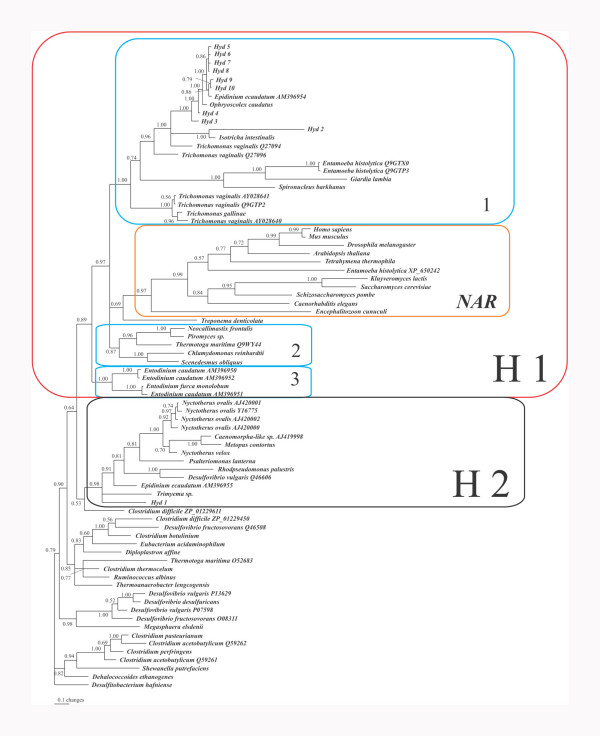
Phylogenetic tree of the H-cluster of [FeFe]-hydrogenases and NARs or NARs-like proteins. Accession numbers of sequences are indicated when more than one sequence from a species is included. The numbers at the nodes represent the posterior probability resulting from a Bayesian inference. *Hyd 1–10*: H-clusters recovered from a metagenomic approach using DNA from total ciliate population in the rumen of a cow. The H1 block marks the "classical " [FeFe] hydrogenases and NAR's. Block 1 is characterized by the clade of *Trichomonas vaginalis *(long and short – type) hydrogenases. It hosts also the majority of the rumen sequences plus the hydrogenases from the type-strain rumen ciliates *Epidinium ecaudatum, Ophryoscolex caudatus*, and *Isotricha intestinalis*. Block 2 marks the long-type hydrogenases from the anaerobic chytridiomycetes *Neocallimastix *and *Piromyces *and the (short) plastidic hydrogenases from the algae *Chlamydomonas *and *Scenedesmus*. Block 3 marks H-clusters from rumen ciliates that are likely to lack hydrogenosomes. Block H2 marks a well supported clade of Fe hydrogenases dominated by *N. ovalis*. Besides *N. ovalis *and its close relatives, this clade consists of hydrogenases from the amoeboflagellate *Psalteriomonas lanterna*, the rumen ciliate *Epidinium ecaudatum*, the free-living ciliate *Trimyema sp*. and the rumen (meta) sequences *Hyd 1*. A fusion of the H-cluster with the 24 and 51 kDa modules has so far only been observed for the *N. ovalis *clade. The *Psalteriomonas *hydrogenase has no fused 24/51 kDa modules.

The phylogenetic analysis of the complete hydrogenase of *N. ovalis *is hampered by the high sequence conservation and the modular organisation of the bacterial and eukaryotic [FeFe] hydrogenases [15,19]. This implicates that only the "H-cluster", a rather small piece of the total hydrogenase, can be used in a phylogenetic analysis that includes all eukaryotic [FeFe] hydrogenases. Also, the very limited sampling of eukaryotic hydrogenases restricts the phylogenetic reconstruction, suggesting an unresolved, deep eukaryotic origin for most of these hydrogenases [13-16]. Therefore, we undertook a metagenomic approach to retrieve hydrogenase-encoding DNA sequence information from the highly diverse and numerous community of anaerobic ciliates thriving in the rumen of cattle, sheep, and goat [25,26]. Using primers directed against conserved regions of the H-cluster (that is shared by all [FeFe] hydrogenases and the functionally unrelated NAR's [15]) we sequenced 10 clones derived from the total rumen ciliate DNA. In addition, we determined the homologous DNA sequences from 9 validated type strains of rumen ciliates. Phylogenetic analysis of this extended data set reveals the existence of two clades of eukaryotic [FeFe] hydrogenases, indicated by H1 and H2 in Figure 4. The H1 clade contains the majority of the eukaryotic sequences, including the NAR'S.

The H2 clade comprises all hydrogenase sequences from *N. ovalis *and its intestinal and free-living relatives. In addition, the H-clusters from two rumen ciliates, one amoeboflagellate, and the ciliate *Trimyema *sp. belong to this clade – besides sequences from the *delta*-proteobacterium *Desulfovibrio vulgaris *and the *alpha*-proteobacterium *Rhodopseudomonas palustris*. A bacterial, i.e. endo/episymbiotic origin of the sequences derived from the two rumen ciliates and the ciliate *Trimyema *cannot be excluded at the current state of information. The ciliate origin of the *N. ovalis *sequences has been confirmed by their assignment to gene-sized macronuclear chromosomes that are characteristic for *Nyctotherus *and its relatives. Furthermore, the codon usage is characteristic for *N. ovalis *(see below). The hydrogenase of the amoeboflagellate *Psalteriomonas lanterna*, on the other hand has recently been recovered from cDNA thereby revealing the absence of C-terminal 24/51 kDa modules (unpublished). Thus, the existence of two different eukaryotic hydrogenase clades is clearly supported, with a clustering of the *N. ovalis *sequence with those from *Desulfovibrio vulgaris *and *Rhodopseudomonas palustris*. The relationship of both clades to other bacterial hydrogenases remains poorly resolved, but there is no evidence for any close relationship to those bacterial taxa that are supposed to be the source for the hydrogenosomal 24/51 kDa modules.(Fig. 2, 3,), indicating an independent origin for the hydrogenase on the one hand and the 24/51 kDa modules on the other hand.

### The [FeFe] hydrogenase of *N. ovalis *is chimeric and has been acquired by lateral gene transfer

As shown above, the 24 kDa and 51 kDa modules of the hydrogenase of *N. ovalis *are neither of mitochondrial nor of *alpha*-proteobacterial origin. Given the presence of paralogues of genuine mitochondrial descent that encode constituents of a functional mitochondrial/hydrogenosomal complex I [11], an acquisition of the whole hydrogenase by lateral gene transfer from different sources is very likely.

**Figure 5:**
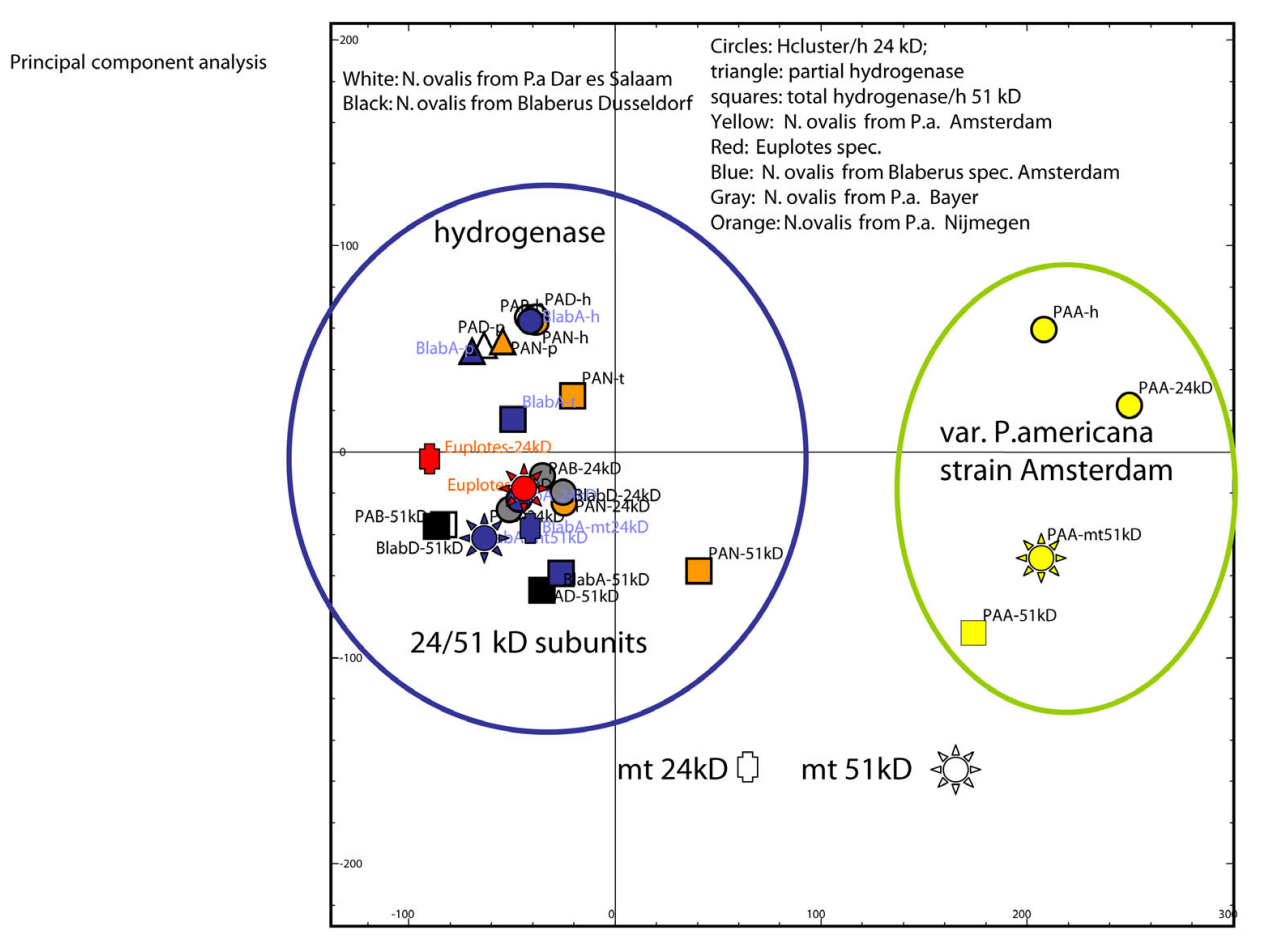
Principal component analysis of the codon-usage of the hydrogenase and mitochondrial 24/51 kDa modules. While most of the *N. ovalis *strains exhibit only slight differences in codon-preference, the isolate *N. ovalis *from the host cockroach *P. americana *strain Amsterdam has a substantially different codon-usage. In both cases, the bacterial-derived 24 and 51 kDa modules acquired the typical ciliate codon-usage that is not significantly different from the one used for the (nuclear-encoded) mitochondrial modules. Even the top-down distribution shows a complete ameliorisation of the modules.

Both the 24 kDa and 51 kDa modules might have been acquired from *beta*-proteobacteria similar to *Rhodococcus *or *Ralstonia*, which possess [NiFe] hydrogenase modules, rather than [FeFe] hydrogenases [19]. Notably, in *Rhodococcus *or *Ralstonia *the 24 kDa and 51 kDa subunits belong to a rather oxygen-resistant [NiFe] hydrogenase, which is not homologous to the [FeFe]-hydrogenases [13-16,18,19,27,28]. An analysis of the codon usage of the various hydrogenase components and their mitochondrial orthologues (Fig. 5) reveals that (1) the hydrogenase modules have acquired the host-specific codon-usage, (2) hydrogenase modules cluster with hydrogenase modules, and (3) mitochondrial genes with mitochondrial genes.

### Why acquire a [FeFe]-only hydrogenase?

We have shown recently that the hydrogenosome of *N. ovalis *is a ciliate-type mitochondrion that produces hydrogen [11]. The presence of a mitochondrial genome, mitochondrial complex I and II dependent respiratory-chain activity, in combination with a kind of fumarate-respiration identifies the *N. ovalis *hydrogenosome as an intermediate stage in the evolution of mitochondria to hydrogenosomes [11,12]. But why acquire a [FeFe]hydrogenase at all? It is likely that the ancestral mitochondrion of *N. ovalis *oxidised NADH via an electron transport chain – as indicated by the presence of genes encoding components of mitochondrial complex I and II. An adaptation to anaerobic environments might be greatly facilitated by the acquisition of a hydrogenase, which could use NADH. It is obvious that the use of fumarate alone as endogenous electron acceptor requires a well-controlled balance between the various catabolic and anabolic reactions in the cell. Depending on the metabolic state of the cell, the NADH pool might be subject to large fluctuations. The presence of alternative oxidases in anaerobic mitochondria provides a means for the cell to cope with such fluctuations in the NADH pool [29]. Such an alternative oxidase appears to be absent in *N*. ovalis, and the hydrogenase could fulfil the task to regulate the NADH pool. The chimeric [FeFe]-hydrogenase of *N. ovalis *is tailored for this requirement since it allows a direct re-oxidation of NADH, due to the presence of the 24 and 51 kDa modules. Other [FeFe] hydrogenases, e.g. those of *Trichomonas vaginalis*, require ferredoxin for hydrogen production from PFO-generated reduction equivalents, and a diaphorase to reoxidise NADH. Thus, a hydrogenase like the one found in *N. ovalis*, provides many advantages for an organism adapting to anaerobic environments. Since there is no evidence for the presence of hydrogenases in mitochondria/protomitochondria [30,31], the scenario for the evolution of the hydrogenosomes of *N. ovalis *from ciliate mitochondria as described here, is likely to have involved complex lateral gene transfer and the fusion of functional domains. The fusion of genes of different origin has at least two advantages: guaranteeing the synthesis of all components in equimolar amounts, and facilitating the flow of electrons from NADH to H^+ ^in a single molecule. Lastly, the evolution of a hydrogenosomal polyprotein requires only the acquisition of a single mitochondrial targeting signal, which can be acquired easily as demonstrated by the frequent retargeting of proteins in the evolution of the eukaryotic cell [31,32].

### Hydrogenases and the origin of mitochondria

The scenario depicted here for the origin of the *N. ovalis *hydrogenase, in which a hydrogenase was added to an aerobic mitochondrion does not necessarily hold for other hydrogenosomes, because they have evolved independently of *N. ovalis *and because they are metabolically less similar to mitochondria than is the *N. ovalis *organelle, e.g. in the way they metabolise pyruvate. *N. ovalis *uses a "mitochondrial" pyruvate dehydrogenase that reduces NAD, which can subsequently be reoxidized by the hydrogenase that has acquired NADH-oxidizing domains. In contrast, the hydrogenosome of *Trichomonas *species metabolise pyruvate via a pyruvate:ferredoxin oxidoreductase[1] and the anaerobic chytrids metabolise it via a pyruvate formate-lyase[33]. The proteins in the hydrogenosomes of anaerobic chytrids are phylogenetically related to the proteins in the mitochondria of aerobic fungi, suggesting also here the evolution of the hydrogenosome as a secondary adaptation to anaerobic circumstances. *Trichomonas*, however, appears in many-sequence based phylogenies at the root of the eukaryotic tree and does not have aerobic, mitochondria containing relatives. A scenario as depicted in the hydrogen hypothesis of Martin and Müller [34], in which the ancestral organelle of all mitochondria and hydrogenosomes had both a respiratory chain and a hydrogenase can therefore not be ruled out. It should thereby be noted that *T. vaginalis*, just like *N. ovalis *has the NADH oxidizing elements of complex I, but, in contrast to *N. ovalis*, does not have the other proteins of this complex [35], and is with respect to complex I more like *Schizosaccharomyces pombe *[21].

## Conclusion

*N. ovalis *acquired its unique [FeFe] hydrogenase by lateral gene transfer from two different sources. Given that *N. ovalis *performs a kind of fumarate respiration allowing survival under anaerobic conditions, the acquisition of this peculiar [FeFe] hydrogenase allows an additional regulation of the NADH pool, which is crucial for maintaining the metabolic homeostasis under anaerobic conditions.

## Methods

### Isolation (and culture) of the ciliates

*N. ovalis *was isolated from the hindgut of the cockroaches *Periplaneta americana *strains Amsterdam (PA), Bayer (PB), Dar es Salaam (PD), Nijmegen(PN) and *Blaberus sp*. strains Düsseldorf (BD) and Amsterdam (BA) taking advantage of the unique anodic galvanotaxic behaviour of *N. ovalis *[36].*Euplotes *sp. was grown in Erlenmeyer flasks containing 500 ml artificial seawater (465 mM NaCl, 10 mM KCl, 53 mM MgCl_2_, 28 mM MgSO_4_, 1.0 mM CaCl_2_, and 0.23 mM NaHCO_3_). Since *Euplotes *sp. requires living bacteria for growth, *E. coli *XL1-blue was supplied at regular intervals. Alternatively, a small piece of beef-steak (approximately 1 cm^3^) was placed into the culture medium to allow the growth of food bacteria. *Euplotes *sp. cells were harvested 28 days after the start of a new culture by filtration through a 4 μm plankton gaze.

Rumen ciliates were isolated by electromigration from the rumen fluid of a grass-fed, fistulated Holstein-Friesian cow, and lysed immediately after the isolation in a 8 M solution of guanidinium chloride and stored at minus 25°C until use.

### DNA isolation, total RNA isolation and cDNA synthesis

DNA of *N. ovalis *and *Euplotes *sp. was isolated according to van Hoek et al. [37]. Total rumen ciliate DNA was prepared after purification on a hydroxyapatite column (BioRad) using standard methods. Total RNA of *N. ovalis *was isolated using the RNeasy Plant mini-kit (Qiagen). Adaptor-ligated cDNA was prepared according to the SMART™ RACE cDNA Amplification kit (Clontech).

Isolation of the H-cluster, the 24 kD (hoxF) and 51 kD (hoxU) modules of the hydrogenase gene, and mitochondrial-type 24 kDa and 51 kDa subunits of mitochondrial complex *I*

*H-clusters of Fe-hydrogenases were amplified from total rumen ciliate DNA using PCR with primers described earlier *[15]. *In addition, DNA from type-strain rumen ciliates, kept by the ERCULE consortium was used as template for PCR*.

To isolate the (nuclear-encoded) *24 kD (hoxF) *and *51 kD (hoxU) *-like genes, the primer-design was based on the H-cluster and the 24 kD (hoxF) and 51 kD (hoxU) region of the hydrogenase of *N. ovalis *PN [18]. Their sequences are 5'-gtnatggcntgyccngghgghtg-3' (H-cluster forward primer) and 5'-ccntcyctrcadggnacrcaytg-3' (51 kDa reverse primer 1). Sequence-specific internal primers were designed to isolate the termini of the gene-sized chromosomes in combination with a telomere-specific primer using the telomere suppression PCR method [38,39].

To isolate the (nuclear) genes encoding the 24 kDa and 51 kDa subunits of mitochondrial complex I, respectively, primers were based on conserved amino-acid regions of mitochondrial complex I genes. Their sequences are 5'-tgyggwachachccwtg-3' (24 kDa forward primer), 5'-ccnarrcaytcdacytc-3' (24 kDa reverse primer), 5'-gmhgargghgarccwgghac-3' (51 kDa forward primer), and 5'-cangwcatytcytcytcnac-3' (51 kDa reverse primer). The ORFs were completed as described above.

### Phylogenetic analysis

The amino acid sequences of the H-cluster were aligned using Clustal × 1.81 [40]. The program Gblocks [41] was used to identify regions of defined sequence conservation and exclude ambiguously aligned positions from the alignment. The phylogenetic analysis of the sequences were performed with the program MRBAYES version 3.1.2 [42]. Markov chain Monte Carlo from a random starting tree was initiated and run for 2 million generations. In these analyses, the JTT model of amino acid substitution and four gamma distributed rates of evolution were applied. Trees were sampled every 1000th generation. The first 25% of the samples were discarded as 'burn-in', and the rest of the samples were used for inferring a Bayesian tree. Examination of the log-likelihood and the observed consistency with the similar likelihood values between the two independent runs suggest that the run reached stationarity and that these burn-in periods were sufficiently long.

The accession numbers of the sequences used to calculate this tree are *Nyctotherus ovalis *BA AY608627; *N. ovalis *PN CAA76373; the sequences from rumen ciliates have been deposited in GenBank under accession numbers AM396939 – AM396957

For the 24kD/51kD domains less sequences had to be included to delineate the evolution, allowing a "manual" sequence alignment and phylogeny approach. Alignments of representative sequences from the 24kD/51kD domains were generated with MUSCLE [43]. Sequences were edited and the most relevant parts from the alignments were selected manually using Seaview [44]. Phylogenies were subsequently derived using the program PHYML [45] using the JTT model and an estimated number of invariable sites with four substitution rate categories. 100 bootstraps were performed; they are only indicated in the tree if they are ≥ 50.

The accession numbers of the used 24 kDa subunit/NuoE/hoxF sequences are [*Agrobacter tumefaciens *PIR:D97514, *Bos taurus *Swiss-Prot:P04394, *Buchnera aphidicola *Swiss-Prot:P57255, *Burkholderia cenocepacia *AU 1054 REFSEQ:YP_621511.1, *Burkholderia xenovorans *LB400 REFSEQ:YP_555778.1, *Dechloromonas aromatica *RCB GenBank:AAZ45735.1, *Deinococcus radiodurans *REFSEQ:NP_295224, *Drosophila melanogaster *GenBank:AAL68189, *Escherichia coli *Swiss-Prot:P33601, *Hahella chejuensis *KCTC 2396 REFSEQ:YP_431454.1, *Heliobacillus mobilis *EMBL:CAJ44288.1, *Homo sapiens *REFSEQ:NP_066552, *Magnetospirillum magneticum *AMB-1 REFSEQ:YP_422756.1, *Methanospirillum hungatei *JF-1 REFSEQ:YP_502735.1, *Methanothermobacter thermautotrophicus *str. Delta H GenBank:AAB86022.1, *Methylococcus capsulatus *str. Bath REFSEQ:YP_115124.1, *Mus musculus *Swiss-Prot:Q9D6J6, *Mycobacterium vanbaalenii *PYR-1 REFSEQ:ZP_01205720.1, *N. ovalis *BA (24 kDa) GenBank:AY628688, *N. ovalis *BD GenBank:AY608628, *N. ovalis *PA GenBank:AY608629, *N. ovalis *PB GenBank:AY608630, *N. ovalis *PD GenBank:AY608631, *N. ovalis *PN GenBank:CAA76373, *Neurospora crassa *Swiss-Prot:P40915, *Nitrosospira multiformis *ATCC 25196 REFSEQ:YP_411790.1, *Nitrosospira multiformis *ATCC 25196 REFSEQ:YP_412360.1, *Nyctotherus ovalis *BA GenBank:AY608627, *Paracoccus denitrificans *Swiss-Prot:P29914, *Paramecium tetraurelia *Swiss-Prot:Q6BFW6, *Psychromonas ingrahamii *37 REFSEQ:ZP_01348563.1, *Ralstonia eutropha *GenBank:AAC06140, *Ralstonia metallidurans *CH34 REFSEQ:YP_583086.1, *Ralstonia solanacearum *UW551 REFSEQ:ZP_00943354.1, *Rattus norvegicus *Swiss-Prot:P19234, *Rhodobacter sphaeroides *ATCC 17029 REFSEQ:ZP_00918830.1, *Rhodococcus opacus *Swiss-Prot:P72304, *Rhodoferax ferrireducens *T118 REFSEQ:YP_525088.1, *Streptomyces avermitilis *MA-4680 REFSEQ:NP_823011.1, *Syntrophus aciditrophicus *SB REFSEQ:YP_461127.1, *Tetrahymena thermophila *Swiss-Prot:Q23LJ5, *Thermoanaerobacter tengcongensis *GenBank:AAM24146, *Thermococcus kodakarensis *KOD1 DDBJ:BAD85803.1, *Thermotoga maritima *REFSEQ:NP_227828, *Thermus thermophilus *Swiss-Prot:Q56221, *Thiobacillus denitrificans *ATCC 25259 REFSEQ:YP_314904.1, uncultured archaeon GZfos26D6 GenBank:AAU83055.1]

The accession numbers of the 51 kDa subunit/NuoF/hoxU sequences are [*Agrobacterium tumefaciens *Swiss-Prot:Q8U6U9, *Aquifex aeolicus *Swiss-Prot:O66841, *Arabidopsis thaliana *Swiss-Prot:Q8LAL7, *Aspergillus niger *Swiss-Prot:Q92406, *Bos taurus *GenBank:AF092131, *Bradyrhizobium japonicum *DDBJ:BAC48402, DDBJ:BAC50177, *Burkholderia xenovorans *LB400 REFSEQ:YP_555778.1, *Candida tropicalis *Swiss-Prot:Q96UX4, *Caulobacter crescentus *Swiss-Prot:Q9A6X9, *Chromobacterium violaceum *ATCC 12472 REFSEQ:NP_900616.1, *Dechloromonas aromatica *RCB GenBank:AAZ45735.1, *Dictyostelium discoideum *REFSEQ:XP_636489.1, *Eubacterium acidaminophilum *EMBL:CAC39230.1, *Euplotes *sp. GenBank:AY608636, *Hahella chejuensis *KCTC 2396 REFSEQ:YP_431454.1, *Heliobacillus mobilis *EMBL:CAJ44288.1, *Leishmania major *Swiss-Prot:Q9U4M2, *Magnetospirillum magneticum *AMB-1 REFSEQ:YP_422756.1, *Methanospirillum hungatei *JF-1 REFSEQ:YP_502736.1, *Methanothermobacter thermautotrophicus *str. Delta H GenBank:AAB86023.1, *Methylococcus capsulatus *str. Bath REFSEQ:YP_115124.1, *Mycobacterium tuberculosis *Swiss-Prot:P95176, *Mycobacterium vanbaalenii *PYR-1 REFSEQ:ZP_01205720.1, *N. ovalis *BA (51 kDa) GenBank:AY608632, *N. ovalis *BD GenBank:AY608628, *N. ovalis *PA (51 kDa) GenBank:AY608635, *N. ovalis *PB GenBank:AY608630, *N. ovalis *PD GenBank:AY608631, *N. ovalis *PN GenBank:CAA76373, *Neisseria gonorrhoeae *FA 1090 REFSEQ:YP_208779.1, *Neisseria meningitidis *Z2491 EMBL:CAB83334.1, *Neurospora crassa *Swiss-Prot:P24917, *Nitrosococcus oceani *ATCC 19707 GenBank:ABA59013.1, *Nitrosomonas europaea *ATCC 19718 EMBL:CAD85683.1, *Nitrosomonas eutropha *C71 REFSEQ:ZP_00669832.1, *Nitrosospira multiformis *ATCC 25196 REFSEQ:YP_411791.1, REFSEQ:YP_412360.1, *Nyctotherus ovalis *BA GenBank:AY608627, *Paracoccus denitrificans *Swiss-Prot:P29913, *Psychromonas ingrahamii *37 REFSEQ:ZP_01348563.1, *Ralstonia eutropha *GenBank:AAC06140, *Ralstonia eutropha *JMP134 GenBank:AAZ60345.1, *Ralstonia metallidurans *CH34 REFSEQ:YP_583087.1, *Ralstonia solanacearum *EMBL:CAD15764.1, *Ralstonia solanacearum *UW551 REFSEQ:ZP_00943355.1, *Rhizobium loti *Swiss-Prot:Q98BW8, Swiss-Prot:Q98KR0, *Rhizobium meliloti *Swiss-Prot:P56912, Swiss-Prot:P56913, *Rhodobacter capsulatus *Swiss-Prot:O07948, *Rhodococcus opacus *Swiss-Prot:P72304, *Rhodoferax ferrireducens *T118 REFSEQ:YP_525088.1, *Rickettsia prowazekii *Swiss-Prot:Q9ZE33, *Solanum tuberosum *Swiss-Prot:Q43840, *Streptomyces avermitilis *MA-4680 REFSEQ:NP_823011.1, *Synechococcus elongatus *PCC 6301 EMBL:CAA73873.1, *Syntrophus aciditrophicus *SB REFSEQ:YP_461127.1, *Tetrahymena thermophila *SB210 GenBank:EAR96899.1, *Thermococcus kodakarensis *KOD1 DDBJ:BAD85803.1, *Thermotoga maritima *Swiss-Prot:O52682, Swiss-Prot:Q9WXM5, Swiss-Prot:Q9WY70, *Thermus thermophilus *Swiss-Prot:Q56222, *Thiobacillus denitrificans *ATCC 25259 REFSEQ:YP_314905.1, *Trypanosoma brucei *REFSEQ:XP_824451.1, *Trypanosoma cruzi *GenBank:EAN82122.1, uncultured archaeon GZfos26D6 GenBank:AAU83054.1, *Yarrowia lipolytica *Swiss-Prot:Q9UUU2]

#### Multivariate Comparative Analysis

The codon usage of the genes investigated in this study was subjected to a multivariate analysis by means of Principal Component Analysis (PCA) to visualise the genetic diversity of the ciliate species. PCA was performed using the GeneMaths XT software package (Applied Maths BVBA, Sint-Martens-Latem, Belgium [46]).

## Authors' contributions

GR, SYMVDS and GWMVDS designed the phylogenetic analyses and performed the computional sequence analysis

AHAMVH and NRME performed the codon-usage analyses

BB, AHAMVH, ES, GWMVDS, TAVA, RMDG, GC, MK, isolated and cloned the various – hydrogenase and complex I – genes.

TM, JPJ, NRME, CJN and PP established ciliate cell lines for DNA extraction

BB, GR, SYMVDS, AHAMVH, NRME, CJN, MAH, and JHPH participated in drafting the manuscript

JHPH and MAH initiated and coordinated the study, and JHPH wrote the manuscript.

All authors read and approved the final manuscript.

## Supplementary Material

Additional File 1 Multiple sequence alignment of the 24/51 kDa modules Click here for file
