# Supplementary material for: The [FeFe] hydrogenase of Nyctotherus ovalis has a chimeric origin
Source: BMC Evol Biol. 2007 Nov 16;7:230. doi: 10.1186/1471-2148-7-230 (PMC2216082; doi:10.1186/1471-2148-7-230)
Supplement: Additional File 1 — Multiple sequence alignment of the 24/51 kDa modules [file 1471-2148-7-230-S1.pdf]

A

```
E. coli NuoE                                                                           MHENQQPQTEAFELSAAEREAIEHEMHHYED-
R. prowazekii NuoE                                                                     MNTKITN--FTFAFDKKNLNLAETIIKKYPPE
N. crassa 24 kDa     MATKLTPFLMRTAVRAATRLSTKPSTIAPVSRACLSISARRPSDTLMVHRNTPDNNPDIPFKFSADNEKVIEEIIKRYPPQ
H. sapiens 24 kDa         MFFSAALRARAAGLTAHWGRHVRNLHKTVMQNGAG---GALFVHRDTPENNPDTPFDFTPENYKRIEAIVKNYPEG
N. ovalis BA 24 kDa                      MLSFILKRTIRPSSFVRMTGYRFSRLVTHHETPENNDDTYFEFTPENYKIINSLLKRYPDN
N. ovalis PA HDG                                                                              YNADESAGLDSILAKYPD-
N. ovalis BD HDG                                                                               YNVDEAAGLDSILARYPK-
N. ovalis PB HDG                                                                               YNVDEAAGLDSILARYPK-
N. ovalis BA HDG                                                                               YNPNEAAGLDEILAKYPK-
N. ovalis PD HDG                                                                               YNSNEAAGLDEILAKYPK-
N. ovalis PN HDG                                                                               YNANEAAGLDEILAKYPK-
R. opacus HoxF                                                                                     MSGDIKAILERNGS-
R. eutropha HoxF                                                                                   MDSRITTILERYRS-
```

[2Fe-2S]cluster N1a of

```
                                                                       C-----C-------------------
E.c. NuoE     —PRAASIEALKIVQKQRG-WVPDGAIHAIADVLGIPASDVEGVATFYSQIFRQPVGRHVIRYCD-SVVCHIN--GYQGIQAALEKKLN
R.p. NuoE     GKRSAILPLLDLAQRQNGGWLHVSAIEYVANMLEMPYMRAYEVATFYTMFNLNPIGKYHIQVCT-TTPCWLR--GSDNIMKICEKKLA
N.c. 24 kDa   YKKAAVMPLLDLGQRQHG-FCSISVMNEVARLLEMPPMRVYEVASFYTMYNRTPVGKFHVQACT-TTPCQLGGCGSDVIVKAIKEHLG
H.s. 24 kDa   HKAAAVLPVLDLAQRQNG-WLPISAMNKVAEVLQVPPMRVYEVATFYTMYNRKPVGKYHIQVCT-TTPCMLR--NSDSILEAIQKKLG
N.o. 24 kDa   YKKSAVLYLLHLAQKQNGNFLTLAAMNKVAKILEMTNLNVYEVAAFYSMFNREKVGKIRLQICG-TTPCLLC--GARDIMKACEDHLG
N.o. PA HDG   -HPQYLLPIVIEETDQKG-YISDPSLVKIAKHVHPYPPQVESILSHYHFFPRKYTSDTHVYLCR-CHNCMMK--GQSKVMQALKERYG
N.o. BD HDG   -HPQYLLPIIIEESDKKG-YISDPSLVKIANHVQMHAPHVESVISHYHFFPRKHTSDTHVYLCR-CHNCMMK—-GQKKVMQAIKEKYG
N.o. PB HDG   -HPQYLLPIIIEESDKKG-YISDPSLVKIANHVQMYAPQVESVISHYHFFPRKHTSDTHVYLCR-CHNCMMK--GQKKVMQAIKEKYG
N.o. BA HDG   -EREYLLPIIIEEHDKKG-YISDPSIVKISEYLGMYPAQIDSILSSYHYFPREHTSDAHVYMCT-CHNCMMK--GQGRLLKTIQETYD
N.o. PD HDG   -EQEYLLPIIIEEHDKKG-YISDPSIVKISEHLGMYPAQIDSILSSYHYFPREHTSDAHVYMCT-CHNCMMK--GQGRLLKTIQETYD
N.o. PN HDG   -EKEYLMPIIIEEHDKKG-YISDPSIVKISEHLGMYPAQIESILSSYHYFPREHTIAILMSICVHCHNCMMK--GQGRLLKTIQETYD
R.o. HoxF     -ERTRLIDILWDVQHLYG-HIPDEVLPQLADELNLSPLDILETASFYHFFHRKPSGKYRIYLSD-TVIAKMN--GYQAVHDSLERETG
R.e. HoxF     -DRTRLIDILWDVQHEYG-HIPDAVLPQLGAGLKLSPLDIRETASFYHFFLDKPSGKYRIYLCN-SVIAKIN--GYQAVREALERETG
```

24 kDa and NuoE

```
              --------------------C---C
E.c. NuoE     IK--PGQTTFDGRFTLLPTCCLGNCDK-GPNMMID---EDTHAHLTPEAIPELLERYK
R.p. NuoE     IK--HKETTKDQKFTLSEIECLGACVN-APVVQIN---DDYYEDLNEAKMEKLIEQYLNEFKSK
N.c. 24 kDa   IK-—QGETTPDGLFTFIEVECLGACAN-APMVQIN---DDYFEDLTPETIKQVLSALKESVTDVSKAPQPGPQS(28)
H.s. 24 kDa   IK--VGETTPDKLFTLIEVECLGACVN-APMVQIN---DNYYEDLTAKDIEEIIDELKAGKIPKPGPRSGRFSC(24)
N.o. 24 kDa   IK--MGGTTKDGMFTLEEVECLGVCAN-APMMQVN---NEKVYEDLTPEIMPEMLEKFRKGEEIKAGPQTKGRK(45)
N.o. PA HDG   VDNLHSSVSRDGKFTFHTMNWLGYCVNDGPAMMVKRRGGDYVEVLTGLTGDGVEQS---LKGLKGNVFKWAKNK
N.o. BD HDG   VQDFHGSVSKNGKFTFHGMNWLGYCVNDGPAMLIKRTGGDYVETLTGLSGDNIEES---LNSLKGKTYKWAKNN
N.o. PB HDG   VQDFHGSVSKNGKFTFHAMNWLGYCVNDGPAMLIKRTGGDYVETLTGLSGDSIEES---LNSLKGKTYKWAKNN
N.o. BA HDG   INKTHGGVAKDGSFTLHTLNWLGYCVNDAPAMMIKRKGTNYVETFTGLLEDNIDQRRKALKDLKKELPKWPKNN
N.o. PD HDG   INKTHGGVAKDGSFTLHTLNWLGYCVNDAPAMMIKRKGTNYVETFTGLLEDNIDQRLKALKDLKKELPKWPKNN
N.o. PN HDG   IHETHGGVAKDGSFTLHTLNWLGYCVNDAPAMMIKRKGTNYVETFTGLLGDNIDQRLKSLKNLKKELPKWPKNN
R.o. HoxF     AR--FGGTDKTGMFGLFETPCIGLSDQ-EPAMLIDNVVFTRLRPGTIVDIITQLRQGRSPEDIANPAGLPSDDV(436)
R.e. HoxF     IR--FGETDPNGMFGLFDTPCIGLSDQ-EPAMLIDKVVFTRLRPGKITDIIAQLKQGRSPAEIANPAGLPSQDI(434)
```

B **NADH binding domain**

```
                                                                          ----G-GG-G---G------------------
E. coli NuoF          (22)PVWLDEYRSKNGYEGA---------RKALTGLSPDEIVNQVKDAGLKGRGGAGFSTGLKWSLMPKDESMNIRYLL
R. prowazekii NuoF    (17)YDLKSSQKRGDWYN-----------TKALIDKGRDFIIDEVKKSGLRGRGGAGFSTGMKWSFMPKNSE-KPCYLV
H. sapiens 51 kDa     (50)WRLKGSLSRGDWYK-----------TKEILLKGPDWILGEIKTSGLRGRGGAGFPTGLKWSFMNKPSDGRPKYLV
Euplotes sp. 51 kDa   (47)PFIDGALKRGDWHK-----------TKDIVLNGDDWIIDQIKASGLRGRGGAGFPSGLKYSFMPKVSDGRPSYLV
N. ovalis PA 51 kDa   (50)PFIDGALKRGDWYR-----------TKDILACPHDFVIDEIKKSGLRGRGGAGFPSGLKYSFMPKKSDGRPSFLV
N. ovalis BA 51 kDa   (49)PFINGALKRGDWYR-----------TKDILSCPQDFVIDEIKKSGLRGRGGAGFPSGLKYSFMPKKSDGRPSYLV
N. ovalis BD HDG          IVEQSLKSKGKEYSLLENHISVKDAIKKAVQMGSMKVIKEVTEAKLLGRGGAGFMTGRKWESAYKADV-KEKYVV
N. ovalis PB HDG          IVEQSLKAKGKEYSLIENHISVKDAIKKAVQMGPMKVIKEVTEAKLLGRGGAGFMTGRKWESAYKADV-KEKYVV
N. ovalis BA HDG          IKEMRSQRDGNGYSCMNTQAPIAEATKKAVSMGPEKVIEEIFKSNLVGRGGAGFRTGKKWESAYKTPA-TDKYVV
N. ovalis PD HDG          IKEMRSQRDGNGYSCMNTQAPIAEATKKAVSMGPEKVIEEIFKSNLVGRGGAGFRTGKKWESAYKTPA-TDKYVV
N. ovalis PN HDG          IREMKSQRNGNSYSCMNTQAPIAEATKKAVSMGPEKVIEEVFKSNLVGRGGAGFRTGKKWESAYKTPA-SDKYVV
R. opacus HoxU       (173)VVESNVRTKGPVFFRGLTDYG--RLLELCLALRPEQIIDRIIESKLRGRGGAGFSTGLKWQLCRTAVS-DDKYII
R. eutropha HoxU     (173)MVESNVRTKGPVFFRGRTDLR--SLLDQCLLLKPEQVIETIVDSRLRGRGGAGFSTGLKWRLCRDAES-EQKYVI

                      --------------
E.c. NuoF             CNADEMEPGTYKDRLLMEQLP--HLLVEGMLISAFALKAYRGYIFLRGEYIEAAVNLRRAIAEATEAGLLGKNI-MGTGFDFE
R.p. NuoF             VNADESEPGTCKDRDILRFEP--HKLIEGCLIASFAIGANTCYIYIRGEFYNEASNMQRALDEAYKEGLIGKNA-CGSGFDCN
H.s. 51 kDa           VNADEGEPGTCKDREILRHDP--HKLLEGCLVGGRAMGARAAYIYIRGEFYNEASNLQVAIREAYEAGLIGKNA-CGSGYDFD
E.sp. 51 kDa          INADESEPGTCKDREIMRHDP--HKLIEGALqVGVSMRAKAAYIYIRGEFWYQYNCLERAISEAYERGLIGKNA-CGTGYDFD
N.o. PA 51 kDa        VNADESEPATCKDREIMRHDP--HKLVEGALIVGYAMRARAAYVYIRGEFWDEAMQLERAVAEAYEKGFIGKNA-CGSGYDFD
N.o. BA 51 kDa        VNADESEPATCKDREIMRHDP--HKLVEGALIVGYAMRAKAAYVYIRGEFWHEAQQLERAVAEAYEKGFIGKNA-CGSGYDFD
N.o. BD HDG           CNADEGLPSTFKDWYLLNNENKRKEVLAGMGICANTIGAKKCYLYLRYEYRNLVPNIEADIKKLQSE--------CPELAMLN
N.o. PB HDG           CNADEGLPSTFKDWYLLNNENKRKEVLAGMGICANTIGAKKCYLYLRYEYRNLVPNIEADIKKLQSE--------CPELAMLN
N.o. BA HDG           CNADEGLPSTYKDWCLLNHEVKRKEVFTGMGICAKTIGAKRCFLYLRYEYRNLVPALEQAIKDVQRT--------CPELADLK
N.o. PD HDG           CNADEGLPSTYKDWCLLNHEVKRKEVFTGMGICAKTIGAKRCFLYLRYEYRNLVPALEQAIMDVQRT--------CPELADLK
N.o. PN HDG           CNADEGLPSTYKDWCLLNNEAKRKEVFTGMGICAKTIGAKRCFMYLRYEYRNLVPALEQSIKDVQST--------CPELADLK
R.o. HoxU             CNADEGEPGTFKDRVLLTRSP--KKVFMGMIIAARAIGSRNGILYLRWEYIYLKDYLERQLQELRDEGLLGARIGGQSGFDFD
R.e. HoxU             CNADEGEPGTFKDRVLLTRAP--KKVFVGMVIAAYAIGCRKGIVYLRGEYFYLKDYLERQLQELREDGLLGRAIGGRAGFDFD
```

FMN binding site

```
                       G-G----G----------G----P---PP----P--------P--------
E.c. NuoF         LFVHTGAGRYICGEETALINSLEGRRANPRSKPP---FPATSGVWGKPTCVNNVETLCNVPAILANGVEWYQNISKSKDAGTK
R.p. NuoF         IYLHRGAGAYICGEETALLESLEGKKGMPRLKPP---FPAGFGLYGCPTTINNVESIAVVPTILRRGASWFAAIGKPNNTGTK
H.s. 51 kDa       VFVVRGAGAYICGEETALIESIEGKQGKPRLKPP---FPADVGVFGCPTTVANVETVAVSPTICRRGGTWFAGFGRERNSGTK
E.sp. 51 kDa      VHVVHGAGAYICGEETGLIESLEGKQGKPRMKPP---FPANAGLYGCPTTVTNVETVAVCPTILRRTPEWFASFGRKNNAGTK
N.o. PA 51 kDa    IYVHRGAGAYICGEETSMLSSIEGVIGKPRMKPP---YPAGTGLYGCPTTVTNVETVSTVPTIMRRGGGWFASFGRKNNHGTK
N.o. BA 51 kDa    IYVHRGAGAYVCGEETSMLSSIEGVVGKPRMKPP---YPAGTGLYGCPTTVTNVETVSTVPTIMRRGGSWFASVGRKNNHGTK
N.o. BD HDG       YEVRLGGGPYVAGEENAQFESIQGSAPIPRKDRPSSVFPTIEGLFFKPTVINNVETFACVPHIVQQGSAAFKTNGLP-----K
N.o. PB HDG       YEVRLGGGPYVAGEENAQFESIQGSAPIPRKDRPSSVFPTIEGLFFKPTVINNVETFACVPHIVQQGSAAFKTNGLP-----K
N.o. BA HDG       YEIRLGGGPYVAGEENAQFESIEGRAPLPRKDRPGNVFPTMEGLFHKPTVINNVETFFAVPHIIQQGSQDFGEGKMP-----K
N.o. PD HDG       YEIRLGGGPYVAGEENAQFESIEGRAPLPRKDRPGNVFPTMEGLFHKPTVINNVETFFAVPHIIQQGSQSFGEGKMP-----K
N.o. PN HDG       YEIRLGGGPYVAGEENAQFESIEGRAPLPRKDRPGNIFPTMEGLFHKPTVINNVETFFAIPHIIQQGSQSFGEGKMP-----K
R.o. HoxU         IRIQMGAGAYICGDESALIESCEGKRGTPRVKPP---FPVQEGYLGKPTCVNNVETFAAAARIMEEGPNWFRALGTPESTGTR
R.e. HoxU         IRIQMGAGAYICGDESALIESCEGKRGTPRVKPP---FPVQQGYLGKPTSVNNVETFAAVSRIMEEGADWFRAMGTPDSAGTR
```

```
E.c. NuoF         LMGFSGRVKNPGLWELPFGTTAREILEDYAGGMRDGLK-FKAWQPGGAGTDFLTEAHLD-LPMEFESIGKAGSRLGTALAMAV
R.p. NuoF         VFCISGHVNKPCNIEEVMGVPLKELIEKYAGGVRGGWNNLKAIIPGGASVPLLPKSLCE-VEMDFDSLRTVGSGLGTGGIIVM
H.s. 51 kDa       LFNISGHVNHPCTVEEEMSVPLKELIEKHAGGVTGGWDNLLAVIPGGSSTPLIPKSVCETVLMDFDALVQAQTGLGTAAVIVM
E.sp. 51 kDa      LFAVSGHVNNPGVFEEEMSIPLRELIEKHCGGVTGGWDNLKAIIPGGSSVPLLPKRICDDVLMDFDALRDVRSGLGTAAVIVM
N.o. PA 51 kDa    LYCLSGHINNPCTVEDEMSISLRELIERHGGGVRGGWDNLLGVIPGGSSTPIISKELCETALMDFDSLIEAGSAFGTAGVIVL
N.o. BA 51 kDa    LYSLSGHINNPCTVEDEMSISVRELIERHGGGVRGGWDNLLGVIPGGSSTPIISKELCETALMDFDSLIEAGSAFGTAGVIVL
N.o. BD HDG       LLSVSGDVERPLIIECLLNG----YTLNDLIKDAKARD-VAAAEIGGCTEPLVFRDKFS--MP-FGFGKNVLNAAG--SVVLF
N.o. PB HDG       LLSVSGDVERPLIIECLLNG----YTLNDLIKDAKARD-VAAAEIGGCTEPLVFHDKFS--MP-FGFGKNVLNAAG--SVVLF
N.o. BA HDG       LLSVTGDVEQPILIETHLNN----YSLNHLLKEIDAKD-IVAAEIGGCTEPIIFGSKFD--TL-FGFGKGTLNAVG--SVVLF
N.o. PD HDG       LLSVTGDVDAPILFETHLNN----YSLNHLLKEIDAKD-IVAAEIGGCTEPIIFGSKFD--TL-FGFGKGTLNAVG--SVVLF
N.o. PN HDG       LLSVTGDVDEPILIETNLNN----YSLNHLLQEISAKD-IVAAEIGGCTEPIIFGSKFD--TL-FGFGRGTLNAVG--SVVLF
R.o. HoxU         LLSVAGDCSRPGIYEVEWGV-----TLNEVLTTVGARD-ARAVQISGPSGQCVSVAEDG--ER--RMAYEDISCNG--AFTIF
R.e. HoxU         LLSVAGDCSKPGIYEVEWGV-----TLNEVLAMVGARD-ARAVQISGPSGECVSVAKDG--ER--KLAYEDLSCNG--AFTIF
```

[4Fe-4S]cluster N3 of 51 kDa and NuoF

```
                                       C--C--C---------------------------------------C
E.c. NuoF         DHEINMVSLVRNLEEFFARESCGWCTPCRDGLPWSVKILRALERGEGQPGDIETLEQLCRFLGPGKTFCAHAPGAVEPLQSAI
R.p. NuoF         DKSTDIIYAIARLSKFYMHESCGQCTPCREGTGWMWRVMMRLVNGNAKKNEIDTLLNVTKEI-EGHTICALGDAAAWPIQGLI
H.s. 51 kDa       DRSTDIVKAIARLIEFYKHESCGQCTPCREGVDWMNKVMARFVRGDARPAEIDSLWEISKQI-EGHTICALGDGAAWPVQGLI
E.sp. 51 kDa      DKSTDVIAAIHRLSKFYAHESCGQCTPCREGTSWMEDMLGRMRKGNADFAEIDMLEELSYQI-EGHTICALGDAAAWPVQGLI
N.o. PA 51 kDa    DKSTDVIDAILRLSKFYLHESCGQCTPCREGTGWLVDILERMKIGKADYGEIDQLYELTKQI-EGHTICALGEAAAWPVQGLI
N.o. BA 51 kDa    DKSTDVIDAILRLSKFYLHESCGQCTPCREGTGWLVDMLERMKIGKADYGEIDQLYELTKQI-EGHTICALGEAAAWPVQGLI
N.o. BD HDG       DTACDFGEVYSNKLHFMADESCKQCVPCRDGAQLLHKAFDQMRTTGKTKYNERSLKTAAEAA-KLSAICAHGKALDPLFDSAC
N.o. PB HDG       DTACDFGEVYSNKLHFMADESCKQCVPCRDGAQLLHKAFDQMRTTGKTKYNERSLKTAAEAA-KLSAICAHGKALNPLFDSAC
N.o. BA HDG       NSSCDLGKIYENKLKFMSEESCKQCVPCRDGSYIFHRAFKELRDTGKSSYNMRALSVASESA-ARSSICAHGKALEGLVKAAF
N.o. PD HDG       NSSCDLGKIYENKLKFMSEESCKQCVPCRDGSYIFHRAFKELRDTGKTSYNMRALSVASESA-ARSSICAHGKALEGLFKAAF
N.o. PN HDG       NSSCDLGKIYENKLKFMAEESCKQCVPCRDGSYIFHRAFKELRDTGKSSYNMRALAVASESA-ARSSICAHGKALESLFKSAC
R.o. HoxU         NTERDLLEIVKDFMQFFVDESCGICVPCRVGNIDLHKKVELVIAGKACQKDLDDVVSWGALV-KKTSRCGLGATSPNPILTTL
R.e. HoxU         NCKRDLLEIVRDHMQFFVEESCGICVPCRAGNVDLHRKVEWVIAGKACQKDLDDMVSWGALV-RRTSRCGLGATSPKPILTTL
```

```
E.c. NuoF         KYFREEFEAGIKQPFSNTHLINGIQPNLLKERW
R.p. NuoF         RHFRDEIEQRIKSFGIA
H.s. 51 kDa       RHFRPELEERMQRFAQQHQARQAAS
E.sp. 51 kDa      RHFRHEMEDRIEDYKAENPERGQRAKVSHLPEHH
N.o. PA 51 kDa    KHYRDVIEDRIDKYHSEHPAAAEEFIISHHPVKGTH
N.o. BA 51 kDa    KHYRPVMEDRMDKYHSQHPVVAEGPFSSHHPVAGSH
N.o. BD HDG       EYLKSKKPL
N.o. PB HDG       EYLKSKKPL
N.o. BA HDG       DFMNKTKPNY
N.o. PD HDG       DFMNKTKPSY
N.o. PN HDG       DFMNKTKPIY
R.o. HoxU         DKFPEIYTKRLRKQKKEALLLSFDLDAALGGYEKALEGLAKEEIK
R.e. HoxU         EKFPEIYQNKLVRHEGP-LLPSFDLDTALGGYEKALKDLEEVTR
```
